# Criminal liability for spreading fake news: a study of Jordanian law in light of international standards

**DOI:** 10.3389/fsoc.2026.1815400

**Published:** 2026-06-16

**Authors:** Khaled Mohammad Khwaileh, Naheda Mohammad Makhadmeh

**Affiliations:** 1 College of Law, University of Khorfakkan, Sharjah, United Arab Emirates; 2 College of Communication and Media, Al Ain University, Al Ain, United Arab Emirates

**Keywords:** criminal liability, digital sovereignty, fake news, freedom of expression, Jordanian Cybercrime Law, networked authoritarianism

## Abstract

This research investigates the criminalization of fake news under the 2023 Jordanian Cybercrime Law, evaluating its implementation within the broader global canon of fake news law analyses. Adopting a qualitative research design that integrates doctrinal content analysis with comparative legal assessment, the study examines how Jordan's regulatory framework navigates the tension between international human rights standards, specifically Article 19 of the ICCPR and domestic security logics. The analysis identifies a shift toward the securitization of digital speech, characterized by: (i) the use of imprecise legal definitions that allow for the criminalization of political criticism; (ii) the imposition of penalties that prioritize state security over individual expression; and (iii) a state-centric interpretation of digital sovereignty. Through analyzing judicial precedents such as Court of Cassation Decision No. 6280/2022, the paper demonstrates how hybrid regimes utilize legalistic justifications to manage digital dissent. The paper concludes by positioning Jordan's legislative evolution as a case of Coordinated Digital Governance, offering a nuanced understanding of how Global South legal systems negotiate international norms against the backdrop of regional political pressures.

## 1 Introduction

The global information ecosystem has been radically reshaped by the rise of social media, giving birth to a new academic and regulatory paradigm often referred to as the fake news law canon. While the proliferation of disinformation poses genuine challenges to social cohesion, the legislative responses, particularly in the Middle East and North Africa (MENA) region, have increasingly moved toward the securitization of the digital space. In Jordan, where over 80% of the population engages with social media (Al-Jalabneh and Safori, 2023), the government's response via (Cybercrime Law No. 17, 2023) represents a pivotal moment in the state's effort to assert control over the national narrative. This research aims to analyze the 2023 Law not merely as a technical failure to meet international benchmarks, but as a complex negotiation between international human rights norms, domestic political pressures, and the logic of regime survival.

Historically, Jordan's digital governance has evolved through a series of restrictive frameworks, yet the 2023 Cybercrime Law marks a significant escalation in the use of legalistic governance to regulate digital speech. While the law is framed as a tool to combat malicious disinformation, its broad language frequently fails to distinguish between verifiable harm and protected expression, such as satire or investigative journalism. This tension highlights a fundamental divergence in the conceptualization of digital sovereignty. While European benchmarks, such as the Digital Services Act (DSA), view sovereignty through the lens of platform accountability and the protection of citizen rights, the Jordanian model aligns with a state-centric paradigm where sovereignty is interpreted as the state's unilateral right to control its information environment (Pearlman, 2022; Gillespie, 2018).

This paper employs a qualitative research design to examine three core dimensions of the 2023 Law: the precision of its legal definitions (notably Article 15), the proportionality of its criminal sanctions, and its alignment with the tripartite test of legality, necessity, and proportionality mandated by Article 19 of the International Covenant on Civil and Political Rights (ICCPR). To avoid the deficit model often found in human rights scholarship, the paper situates these legal texts within the theoretical frameworks of securitization and Coordinated Digital Governance. This approach recognizes that the ICCPR framework is not a neutral benchmark but a normative standard that hybrid regimes like Jordan must navigate while addressing local histories of media regulation and state security (Jones, 2022). Through the analytical lenses of securitization and networked authoritarianism, this paper evaluates how the Jordanian 2023 Law is utilized not merely for public safety, also to assert state power and manage digital dissent.

Through integrating comparative insights from regional peers like Tunisia and global benchmarks like the EU and Germany, this research fills a critical gap in the literature regarding the 2023 Jordanian legislation. It moves beyond a compliance-based model to ask deeper questions about how hybrid governance regimes utilize the fake news discourse to permanentize emergency-style information controls. Ultimately, the study provides an analytical framework for understanding the evolution of digital governance in the Global South, offering generalizable outcomes for the study of legal pluralism and the politics of rights-based assessments in the digital age.

## 2 Theoretical framework: the fake news law canon and the securitization of information

This research addresses the conceptual challenge of “information disorder” (Wardle and Derakhshan, 2017) by positioning itself within the burgeoning academic canon of fake news law analysis, which examines how states across the globe have responded to this phenomenon through legislative intervention (Bradshaw et al., 2025). While much of the existing literature focuses on either established Western democracies or strictly authoritarian regimes, this paper contributes to the analysis of the hybrid governance paradox found in hybrid regime contexts like Jordan. What distinguishes this study from previous analyses is its focus on the 2023 Cybercrime Law as a manifestation of “authoritarian legality” process where the state utilizes formal legal institutions and the rhetoric of protecting truth to achieve illiberal ends.

### 2.1 Securitization theory and the construction of existential threats

The primary lens used to decode Jordan's regulatory logic is securitization theory. According to this framework, an issue is securitized when a state actor successfully frames a specific phenomenon, in this case, digital disinformation, as an existential threat to national security or social peace (Buzan et al., 1998). Through moving false news from the realm of normal political discourse into the realm of emergency security, the Jordanian state justifies extraordinary measures that would otherwise be considered violations of constitutional free speech. As seen in Articles 15 and 21 of the 2023 Law, the lack of precise legal definitions allows the state to categorize political criticism as a security threat, thereby legitimizing the use of criminal sanctions against social media users (Jones, 2022).

### 2.2 Competing paradigms of digital sovereignty

A central contribution of this framework is the clarification of digital sovereignty, a concept that has evolved significantly and often confusingly in global discourse. In the European Union context, most notably within the Digital Services Act (DSA), digital sovereignty refers to the protection of citizens' data, the accountability of Big Tech, and the preservation of democratic autonomy through platform regulation. In contrast, this paper employs the term in the sense used by Pearlman (2022) to describe the MENA regional paradigm: state-centric digital sovereignty. Here, sovereignty is not about protecting the user from the platform, but about the state asserting unilateral control over the national information space to exclude foreign or subversive narratives. While the EU model emphasizes co-regulation and transparency, Jordan's 2023 Law exemplifies a state-dominant model where digital sovereignty is used as a legalistic shield for regime security.

### 2.3 Networked authoritarianism and legalistic justification

The study further engages with the concept of networked authoritarianism (MacKinnon, 2012). In Jordan's hybrid regime, networked authoritarianism does not rely on crude, total censorship (such as a total internet shutdown). Instead, it operates through a sophisticated combination of nominal democratic institutions and restrictive digital controls. The 2023 Law provides the necessary legalistic justification for this model, allowing the state to maintain a facade of the rule of law while selectively enforcing vague provisions against influential digital voices. Through defining the state as the sole arbiter of truth, the law effectively channels digital dissent into safer, state-sanctioned outlets. This framework of networked authoritarianism is essential for the subsequent discussion, as it explains how Jordan occupies a middle ground between liberal platform governance and digital control.

### 2.4 The legal architecture of control

These theoretical lenses are applied directly to the primary legal instruments: Jordan's 2023 Cybercrime Law (No. 17). Specifically, Article 15 criminalizes the dissemination of false news threatening national security without providing a definition for either term, while Article 21 introduces enhanced penalties for financially motivated disinformation. When read alongside Articles 130–132 of the Penal Code, which historically addressed wartime propaganda, a clear shift emerges. The 2023 framework expands the state's reach from emergency wartime powers into the everyday governance of digital speech, effectively permanentizing the securitization of the space of information.

## 3 Literature review

The regulation of digital rights in the Middle East and North Africa (MENA) has shifted from a focus on liberation technology to a robust scholarly inquiry into what Jones (2022) defines as digital authoritarianism. This evolution is characterized by the use of disinformation, deception, and social media manipulation to maintain regime stability. While early research by Deibert and Rohozinski (2010a,b) identified cyber authoritarianism as a method of dissent suppression, contemporary scholarship suggests that these legal frameworks have matured into a specialized canon of fake news laws (Jones, 2022). These laws often use the legitimate concern of misinformation to justify broad state intervention in digital spaces (Douai, 2019). Reviewer critiques of international legal scholarship often highlight a deficit model where Global South legal systems are assessed primarily by their failure to mirror Western liberal benchmarks. To avoid this, it is necessary to engage with critical human rights theorists and Global South legal scholars who argue that frameworks like the International Covenant on Civil and Political Rights (ICCPR) carry normative assumptions about state authority and individual rationality that may require contextualization in postcolonial or hybrid political contexts (Mutua, 2001). This perspective is furthered by scholars of Arab legal pluralism and postcolonial legality who suggest that law in the region often functions not as a neutral arbiter but as a site of negotiation between inherited colonial structures and contemporary state survival strategies (Shahar, 2012). As Douai (2019); Khwaileh (2025) notes, the post-truth era in the Arab world is not merely a technical problem of misinformation but is deeply tied to the historical politics of media control and regime survival. In Jordan, Al-Jalabneh and Safori (2023) observe that Facebook has become a primary news source, creating a disinformation laboratory where the state's reaction, the 2023 Cybercrime Law, must be understood as a negotiation between international norms and local security logics.

A critical differentiator in recent scholarship is the definition of digital sovereignty. While the European Union's Digital Services Act (DSA) views sovereignty as the protection of citizens' data and platform accountability, Arab states often interpret digital sovereignty as the state's unilateral right to control the national information environment (Pearlman, 2022). This state-centric model is facilitated via networked authoritarianism, a concept popularized by MacKinnon (2012) and expanded through Byman (2021). In this framework, the state does not simply censor the internet but instead uses legal ambiguity and social media wars to dominate the narrative. Byman (2021) argues that social media in the MENA region has transitioned from a tool of protest to a tool of regime resilience where cybercrime laws serve as the legal infrastructure for this transition. Scholarship specific to Jordan has documented a consistent tightening of the digital space. Early analysis of the 2015 Cybercrime Law highlighted how vague provisions created a chilling effect on expression (Freedom House, 2020). However, the 2023 amendments represent a significant escalation. Unlike the soft regulation seen in some liberal contexts, Jordan's approach emphasizes criminal liability for users rather than platform accountability (Hammouri et al., 2024). This aligns with what call authoritarian legality where the law is used as a tool of governance rather than a check on power.

Despite the growing body of literature on digital authoritarianism and regional media policy, a significant research gap remains regarding the practical intersection of these theoretical frameworks with specific judicial application in hybrid regimes. Current scholarship tends to focus either on the broad political implications of cyber laws or on abstract compliance with international human rights benchmarks. There is a lack of granular analysis exploring how specific judicial precedents, such as the rulings of the Jordanian Court of Cassation, bridge the gap between the rhetoric of protecting truth and the reality of securitizing political speech. Furthermore, few studies have examined how the 2023 Jordanian law attempts to synthesize the language of international standards (such as the ICCPR) with the operational mechanisms of networked authoritarianism. This paper fills this gap by moving beyond a simple compliance model to analyse whether Jordan's latest provisions represent a unique hybrid regulatory model designed to manage the fake news threat while maintaining global legal legitimacy.

## 4 Methodology

This paper adopts a qualitative legal research design that integrates doctrinal legal analysis with a systematic comparative assessment of international human rights benchmarks. Rather than performing a simple binary compliance check, the methodology is designed to situate Jordan's Cybercrime Law No. 17 of 2023 within the hybrid governance paradox. Via bridging theoretical legal frameworks with the enforcement realities of digital speech, this approach provides a robust framework for understanding the tension between security imperatives and fundamental digital rights in the MENA region.

The primary data corpus consists of purposively selected national legislation and judicial precedents chosen for their direct impact on information control. Specifically, the study focuses on Articles 15 and 21 of the 2023 Cybercrime Law to examine their deviation from earlier frameworks and international standards. A critical component of the analysis is the review of recent (Court of Cassation, 2022) case law, specifically Decision No. 6280/2022. This case was chosen because it represents the pinnacle of judicial interpretation of fake news in Jordan, illustrating how textual ambiguity can be exploited to silence dissenting voices. Also, its selection because it represents a definitive judicial interpretation of Article 15(a) concerning allegations of public corruption, demonstrating how legal ambiguity is applied in practice.

This focus on case law ensures that the findings emerge from an analysis of practical enforcement rather than a purely descriptive policy review.

To provide both regional and global context, the research utilizes a functional comparative approach by selecting three distinct regulatory models: Tunisia, the European Union (EU), and Germany. Tunisia's Decree-Law No. 54 (2022) (Republic of Tunisia, 2022) was selected as a regional peer to offer a global south perspective on the challenges of balancing disinformation with human rights in post-revolutionary contexts (ARTICLE 19, 2023). Conversely, the EU's Digital Services Act (2022) and Germany's Network Enforcement Act (NetzDG) serve as benchmarks for the liberal democratic model. The EU and Germany were selected not as universal ideals, but because they represent the Brussels Effect, setting global, normative regulatory standards for digital platforms. Comparing Jordan against these models reveals the limits of exporting Western-centric compliance frameworks into hybrid regimes, where local security logics override international normative expectations.

All these models were selected because they emphasize platform accountability, transparency, and tiered sanctions, providing a necessary contrast to Jordan's state-dominant model of user criminalization (Heldt, 2019).

The analytical procedure follows the tripartite test established under international human rights law, legality, necessity, and proportionality, to evaluate the precision of Jordan's legal definitions. To avoid a deficit model that views Global South legal systems solely through the lens of Western failure, this study employs the theoretical lenses of securitization and networked authoritarianism. This reflexive approach acknowledges that the current legislation is not merely a technical failure of precision but is an active negotiation between international norms and domestic security logics. Through identifying specific legislative gaps, such as the absence of a public interest defense, the study provides a roadmap that reconciles security objectives with constitutional and international obligations.

## 5 Jordan's legal framework on fake news

The Jordanian legal approach to combating fake news, primarily enshrined in the Cybercrime Law No. 17 of 2023, exemplifies the tension between national security objectives and international free expression standards (11). The law's broad provisions, particularly Articles 15 and 21, criminalize the dissemination of false news without clear definitions or safeguards for protected speech. Article 15(a) penalizes intentionally sharing content that threatens national security or social peace with fines up to 20,000 JOD (US$28,000) and imprisonment, while Article 21 targets financially motivated disinformation with even harsher sanctions. Critics argue that such vague terminology, failing to distinguish satire, parody, or investigative journalism from malicious disinformation, creates a chilling effect on digital discourse. Where this ambiguity starkly contrasts with international benchmarks like Article 19 of the ICCPR, which mandates that restrictions on speech be precisely defined, necessary, and proportionate. The Jordanian law's overreach is further highlighted by its punitive disparity with the Penal Code's older defamation statutes, which impose negligible fines (max. 50 JOD) for analogous offline speech, suggesting discriminatory treatment of online expression.

Thus, the enforcement of these provisions reveals their potential for misuse. A landmark case, Court of Cassation Decision No. 6280/2022, underscores this risk: a Twitter user was convicted under Article 15(a) for posting corruption allegations against a public official, despite citing verifiable public records. The court's conflation of criticism with false news illustrates how the law's ambiguity enables arbitrary prosecutions. In addition, this pattern aligns with reports by Human Rights Watch (2023), which documented 12 prosecutions in 2023 alone for tweets criticizing government policies, often under the pretext of protecting social peace.

This paper argues that hypothetical scenarios may further reveal the law's flaws, for example, a journalist tweeting about unreported virus deaths could face prosecution, even if they acted in good faith, given the absence of a public interest argument. These cases reflect regional trends, as evidenced by Egypt's use of similar cybercrime laws to silence dissent, but they contrast sharply with Tunisia's Law No. 54 (2022), which requires proof of bad faith and exempts good faith reporting.

Consequently, the law's disproportionate penalties exacerbate its threats to free expression. While the EU's Digital Services Act (2022) and Germany's Network Enforcement Act (2017) employ tiered sanctions based on harm severity, Jordan's blanket penalties, including imprisonment for vague offenses, risk chilling legitimate discourse. Journalists and activists face particular vulnerability, as evidenced by the Jordanian Press Association (2023) finding of a 40% decline in critical online reporting since the law's enactment (Freedom House, 2023). This repression is compounded by Article 15's joint liability for platforms, pressuring social media companies like Twitter/X to preemptively censor contentious content. The lack of procedural safeguards, such as independent judicial review or transparency in government takedown requests, further deviates from global standards like the EU Code of Practice on Disinformation, which mandates platform accountability and user appeals (Deibert, 2019; European Regulators Group for Audiovisual Media Services (ERGA), 2022).

Comparative analysis underscores Jordan's misalignment with international norms. Tunisia's Decree-Law 54, for example, balances disinformation penalties with explicit protections for satire and judicial oversight, while the EU Court of Justice (Case C-18/18, 2019) narrowly defines illegal content to exclude ambiguous or satirical speech. Jordan's framework, by contrast, lacks such nuance, reflecting what scholars' term networked authoritarianism, using vague laws to maintain control while preserving democratic facades. This disconnect is particularly glaring given Jordan's obligations under the ICCPR, which permits speech restrictions only when they meet strict necessity and proportionality tests. The Rabat Plan of Action (32) further emphasizes that laws targeting expression must demonstrate a direct incitement to harm, a threshold Jordan's broad provisions fail to meet.

To align with international standards, Jordan's legal framework requires substantive reforms. Narrowing the definition of false news to require verifiable falsity and malicious intent, as in Tunisia's model, would mitigate overreach. Introducing tiered penalties, reserving imprisonment for cases of provable violence incitement, and adding a public interest defense would better balance security and free expression. Procedural safeguards, such as mandatory judicial review of prosecutions and platform transparency reports, could further prevent abuse. Below, a comparative table summarizes these gaps and proposed solutions: the table comparing key provisions of Jordan's law with ICCPR requirements, EU Code of Practice (2022), Tunisia Decree-Law 54 (2022) and Germany NetzDG (2017).

The comparative analysis reveals that Jordan's current legal framework for combating fake news remains the most punitive among democratic models, particularly due to its harsh prison terms and broad, ambiguous definitions. In contrast, the EU, German, and Tunisian approaches demonstrate a more balanced strategy, emphasizing clearer harm thresholds, platform cooperation over user criminalization, and robust transparency mechanisms (Najjar, 2008). Across all comparator models including Germany's NetzDG-Jordan lags behind in definitional precision, proportionality, and procedural safeguards. To align with international standards while effectively addressing disinformation, Jordan should adopt EU-style verifiable harm thresholds, introduce German-inspired tiered penalties, implement Tunisian judicial oversight mechanisms, and incorporate ICCPR-required public interest defenses. These reforms would help Jordan reconcile its security objectives with its obligations to protect freedom of expression in the digital age.

## 6 Alignment with international human rights standards and proposed reforms

The Jordanian Cybercrime Law No. 17 of 2023, specifically its provisions criminalizing the dissemination of false news, represents a complex negotiation between domestic security imperatives and the normative expectations of international human rights law. While the state-centric regulatory model adopted by Jordan aims to protect public order, its current statutory formalism presents significant challenges regarding compliance with Article 19 of the ICCPR.

Under the ICCPR, the right to freedom of expression is not absolute, but any restriction must survive a rigorous tripartite test: it must be (i) provided by law (legal certainty), (ii) necessary to achieve a legitimate aim, and (iii) proportionate to that aim. In hybrid governance frameworks, the tension between these standards and local security logic often results in a securitization of digital speech. As synthesized in Table 1, the divergence between the 2023 legislative text and these global benchmarks suggests that Jordan's current approach prioritizes immediate information control over the long-term resilience of a pluralistic digital public sphere.

**Table 1:** Comparative analysis of the Jordanian cybercrime law (2023) and international human rights benchmarks (ICCPR Article 19) (Created by the authors).

Key aspect	Jordan's cybercrime law (2023)	ICCPR Article 19 requirements	EU code of practice (2022)	Tunisia decree-law 54 (2022)	Germany NetzDG (2017)
Definition of prohibited content	Vague false news with no distinction from satire/opinion (Art. 15)	Must be precise and narrowly tailored	Focuses on verifiable disinformation with potential public harm	Distinguishes false information from legitimate criticism	Targets only unlawful content (e.g., hate speech, defamation)
Intent requirement	No explicit requirement for malicious intent	Requires demonstration of intent to cause harm	Emphasizes intentional manipulation campaigns	Requires proof of wilful dissemination	Distinguishes negligent vs. intentional sharing
Penalties	3 month−3 years imprisonment + fines up to 20,000 JOD (US$28000)	Must be proportionate to harm	No criminal penalties; platform fines up to 6% global revenue	Fines up to $1,000 + content removal	Tiered fines up to €5 million
Public interest defense	Absent	Required for protected speech	Explicit protections for whistleblowing	Judicial discretion for public interest content	Media exemptions for journalistic content
Platform liability	Joint liability for users and platforms (Art. 15)	Intermediaries should not face disproportionate liability	Good faith protections for compliant platforms	Platforms must appoint local legal representatives	24-h takedown requirement for illegal content
Due process safeguards	Limited judicial oversight pre-prosecution	Requires independent review	Independent audit mechanisms	Specialized cybercrime judges	Court review before content removal
Transparency requirements	None for government actions	Essential for accountability	Mandatory platform transparency reports	Judicial transparency in rulings	Biannual platform compliance reports
Compliance with ICCPR 3-Part test	Fails on precision and proportionality	(Benchmark Standard)	Fully compliant	Partially compliant (improving via reforms)	Compliant after 2021 amendments

A primary concern is the ambiguity inherent in Article 15(a), which criminalizes false news threatening national security or social peace without providing precise definitions for these terms. This lacks the legal certainty required by international law, as it fails to provide citizens with a clear understanding of what constitutes a criminal act. The implications of this ambiguity are illustrated in Court of Cassation Decision No. 6280/2022, where a user was prosecuted for a tweet involving publicly available data. Such cases demonstrate how broad interpretations of false news can expand the zone of criminal liability, contradicting the Rabat Plan of Action, which mandates that speech restrictions be reserved for clear incitement to hostility or violence (Office of the High Commissioner for Human Rights (OHCHR), 2012).

Furthermore, the problematic scope of Article 21, which penalizes financially motivated disinformation, illustrates a failure to distinguish between malicious disinformation campaigns and legitimate digital activities. Without a clear distinction, this provision risks creating a chilling effect on investigative journalism and whistleblowing, where the monetization of reporting could be misinterpreted as criminal intent. Similar patterns of overreach have been documented in other regional models, where vague fake news provisions are utilized to manage dissent through legalistic means.

### 6.1 Proportionality in sanctions

A hallmark of the fake news law paradigm in state-centric systems is the imposition of disproportionate penalties. Jordan's law allows for up to 3 years of imprisonment and fines reaching 20,000 JOD (approx. $28,000). To put this in perspective, these sanctions often surpass those for physical crimes, such as simple assault. According to the UN Human Rights Committee (2011), restrictions must be the least intrusive instrument available to achieve the desired protective outcome.

Through contrast, the European Union's Digital Services Act (DSA) offers a model of risk mitigation rather than individual punishment. While the EU model focuses on platform-level accountability and Scalable Content Moderation, Jordan's framework remains focused on individual criminalization. This punitive approach, as noted by Amnesty International (2023), often deters not only malicious actors but also legitimate social commentary and satire, which are essential components of a healthy democratic discourse.

### 6.2 Strengthening due process and judicial oversight

To reconcile Jordan's framework with international standards, the state must move beyond a purely punitive model toward a procedural safety framework. This requires establishing robust judicial filtering mechanisms. In Germany, for instance, the Network Enforcement Act (NetzDG) requires that any prosecution for online speech undergo a rigorous assessment by independent public prosecutors and, in many cases, judicial authorization before charges is filed (Federal Ministry of Justice, 2017).

Introducing similar mandatory judicial review panels in Jordan would ensure that false news cases are assessed based on legal thresholds of verifiable harm rather than political sensitivity. Via placing the burden of proof on the state to demonstrate both malicious intent and actual public harm, Jordan can protect its citizens from arbitrary enforcement. Such oversight would transform the current model of statutory formalism into a system that upholds the rule of law and protects the judiciary from being used as a tool for managing public criticism.

### 6.3 Platform accountability and transparency

Rather than asserting unilateral control, Jordan should consider a model of co-regulation that emphasizes transparency. The EU Code of Practice on Disinformation (2022) and the DSA provide blueprints for this: they mandate that platforms disclose government takedown requests and provide insights into the algorithms used for content moderation (European Commission, 2020).

Jordan could establish a Digital Transparency Unit to act as an independent liaison between the state, social media platforms, and civil society. Through requiring regular transparency reports, including the legal basis for content removal and the nature of government requests, the state can foster institutional accountability. This approach acknowledges the state's legitimate interest in curbing harmful content while providing the public with the procedural fairness guaranteed by the ICCPR.

### 6.4 Fostering public resilience through media literacy

The most sustainable defense against disinformation is not criminalization, but a digitally literate citizenry. While legal reforms are necessary, they must be paired with efforts to empower the public to critically assess information. UNESCO's think before sharing initiative (2021) provides a global model for this, demonstrating that education is more effective at neutralizing conspiracy theories than censorship.

Jordan could adopt Safe Harbor provisions (similar to Section 230 of the U.S. Communications Decency Act, 1996) to protect platforms acting in good faith, while simultaneously integrating media literacy into national school curricula. This holistic response addresses the root cause of information disorder without infringing upon fundamental freedoms.

### 6.5 Societal implications and the state rationale: balancing sovereignty and rights

This paper concludes that while the 2023 Law was designed to secure the digital environment, its current application has profound societal repercussions. The discouraging effect on digital participation is particularly evident among journalists and activists, where the high cost of legal defense serves as a form of legal harassment. This has led to a shrinking of the public sphere, as critical discourse moves from public platforms like Facebook and X to private, encrypted channels (Jbour, 2023).

However, to maintain an objective analysis, one must acknowledge the state's perspective on digital sovereignty. In a region defined by geopolitical volatility, the Jordanian government views the digital vacuum as a space where misinformation can incite immediate civil unrest or economic instability (Cybercrime Law No. 17 of 2023). From this viewpoint, the law is a necessary shield for public order. External actors aim the state's pursuit of a digital standard of truth at preventing the weaponization of social media.

The core legal critique, therefore, is not directed at the state's right to protect its people, but at the choice of methods. Through shifting from a model of individual criminalization to one based on proportionality, judicial oversight, and media literacy, Jordan can achieve its security objectives without compromising the fundamental rights that underpin its constitutional and international obligations.

## 7 Conclusion

This research has evaluated the Jordan's Cybercrime Law No. 17 of 2023, situating it within the global fake news law paradigm and the broader evolution of state-centric digital governance. Through a qualitative research design, the analysis reveals that while the legislation is framed as a necessary tool for maintaining public order, its current architecture relies on a model of statutory formalism that presents significant challenges to the fundamental right of freedom of expression. Through examining the shift toward the securitization of the digital public sphere, this paper concludes that the law's broad provisions and severe penalties fail to meet the tripartite test of legality, necessity, and proportionality mandated by Article 19 of the ICCPR.

The findings identify three systemic deficiencies that undermine the law's stated objectives. First, the ambiguity inherent in Article 15 exemplified by judicial applications in cases such as Court of Cassation No. 6280/2022, fails to provide the legal certainty required to distinguish protected criticism from malicious disinformation. Second, the imposition of disproportionate criminal sanctions, including significant fines and imprisonment, creates a pervasive chilling effect that discourages investigative journalism and public interest reporting. Third, the current framework reflects a state-centric interpretation of digital sovereignty that prioritizes unilateral information control over the procedural safeguards and platform accountability models seen in contemporary benchmarks like the European Union's Digital Services Act.

The comparative analysis demonstrates networked authoritarianism in action: by utilizing ambiguous phrasing (Article 15), the state securitizes the digital sphere, moving fake news from a regulatory issue to an existential national security threat.

To bridge the divide between domestic security logics and international human rights obligations, this paper proposes a robust reform agenda grounded in procedural justice and comparative best practices. This roadmap necessitates a transition from a purely punitive model to a rule-based stability framework. Key recommendations include:

- **Refining legal definitions:** restricting criminal liability to demonstrably false statements disseminated with specific malicious intent to cause public harm, while explicitly exempting satire, opinion, and good faith reporting.
- **Implementing judicial filtering:** establishing independent judicial oversight mechanisms to review speech-related cases before prosecution, ensuring that the law is not utilized for selective enforcement against digital dissent.
- **Fostering co-regulation and literacy:** adopting transparency requirements for social media platforms and investing in nationwide media literacy initiatives to build societal resilience against disinformation from the bottom up.

The implications of these findings extend beyond the Jordanian context, offering a template for analyzing how hybrid governance frameworks in the Global South negotiate international norms. Via moving away from a deficit model and instead addressing the underlying pressures of digital governance, Jordan could reconcile its security imperatives with its constitutional commitments. The path forward lies in a holistic approach that recognizes that the most sustainable defense against the information disorder is not the restriction of speech, but the strengthening of the rule of law and the protection of a pluralistic, participatory digital public sphere.

Future research should continue to monitor the enforcement patterns of the 2023 Law, particularly through empirical engagement with the judiciary and legal practitioners. As emerging technologies such as generative AI continue to complicate the digital landscape, the need for a rights-respecting, procedurally sound regulatory framework will only become more paramount. Ultimately, refining the Cybercrime Law is not merely a matter of legal compliance; it is an essential step toward ensuring that the digital age strengthens, rather than weakens, the social contract and the integrity of the information environment.
